# Supplementary material for: Learning curve and surgical outcome of robotic assisted colorectal surgery with ERAS program
Source: Sci Rep. 2022 Nov 29;12:20566. doi: 10.1038/s41598-022-24665-w (PMC9709162; doi:10.1038/s41598-022-24665-w)
Supplement: Supplementary file 1 — Supplementary Information 1. [file 41598_2022_24665_MOESM1_ESM.zip › LSC Raw data-Ñ[▒K/057/P(3)_ñw╝╨░O▒Kñσ.pdf]

[redacted]

Pathologic diagnosis:
1. Rectum, laparoscopic low anterior resection --- Adenocarcinoma, moderately differentiated, arising in a tubular adenoma. Tumor invaded to submucosa. The bilateral cut ends and perirectal lymph nodes (0/15) are free of tumor.
2. IMA root, dissection --- Unremarkable (0/7).
3. Colon, cut end, distal, resection --- Unremarkable.

Ancillary study for diagnosis:
1. IHC stains for MLH1, MSH2, PMS2 and MSH6 are done on section G. All above markers are preserved.
2. Deep cut done on section G.

Prognostic and predictive factor:
1. Tumor size: 1.5x1.5 cm.
2. Depth of invasion: Submucosa.
3. Histologic grade: G2: Moderately differentiated.
4. Angiolymphatic invasion: Not identified.
5. Perineural invasion: Not identified.
6. Tumor deposits (discontinuous extramural extension): Not identified.
7. Surgical Margins: a) Proximal Margin: Uninvolved by invasive carcinoma, distance of tumor from margin: 12 cm. b) Distal Margin: Uninvolved by invasive carcinoma, distance of tumor from margin: 1.5 cm. c) Radial or Mesenteric Margin: Uninvolved by invasive carcinoma, distance of tumor from margin: 2.3 cm.
8. Lymph node status: Uninvolved (0/22).
9. Extranodal involvement: Not identified.
10. Tumor regression grading S/P CCRT: No known presurgical therapy.
11. Type of polyp in which invasive carcinoma arose: Tubular adenoma.
12. Additional pathologic findings: Not identified.
13. Pathological TNM Stage: pT1N0(According to the eighth edition, 2017, American Joint Committee on Cancer Staging Guidelines for Tumors / 2017 CAP guideline).
14. TNM descriptors: Not applicable.

Gross description:
The specimen consists of 1) a segment of colon, 15 cm in length and 2 cm in proximal cut end circumference, 3.5 cm in distal circumference, and perirectal soft tissue. A gray tan firm annular elevated and centrally ulcerative tumor, 1.5x1.5x1 cm, is noted, 1.5 cm to the nearest cut end. On cross section, the tumor invades to the submucosa with 2.3 cm to the radial margin. The both cut ends are free of tumor grossly. 2) IMA root, a piece of tan yellow soft tissue, 5.5x4x1.5 cm. 3) distal cut end, a piece of tan mucosa-covered soft tissue, 2x1.5x1 cm. Representative part for section: A-B) specimen 2 C) specimen 3 all for section D-E) both cut end F-G) tumor with CFM H) normal colon J-L) lymph nodes.

Note:
1. The interpretation of MMR panel and recommended studies are as below: (1) No loss of nuclear expression of MMR proteins: low probability of microsatellite instability-high (MSI-H) . (2) Please note: There are exceptions to the above IHC interpretations. These results should not be considered in isolation, and clinical correlation with genetic counseling is recommended to assess the need for germline testing.
2. This case has been peer reviewed by two doctors.
#T-59300_2 #M-82633_2 2 1105 B0000F
#T-C4520_2 #M-09183_2 0 1105 000000

已電子簽章

請病患妥為保管，若不慎遺失需自負個人資料外洩責任！
